# Mitochondrial determinants of chemoresistance

**DOI:** 10.20517/cdr.2019.46

**Published:** 2019-09-19

**Authors:** Ansooya Bokil, Patricia Sancho

**Affiliations:** IIS Aragon, Hospital Universitario Miguel Servet, Zaragoza 50009, Spain.

**Keywords:** Mitochondria, chemoresistance, oxidative phosphorylation, fatty acid oxidation, metabolism, uncoupling proteins, ABC transporters

## Abstract

Chemoresistance constitute nowadays the major contributor to therapy failure in most cancers. There are main factors that mitigate cell response to therapy, such as target organ, inherent sensitivity to the administered compound, its metabolism, drug efflux and influx or alterations on specific cellular targets, among others. We now know that intrinsic properties of cancer cells, including metabolic features, substantially contribute to chemoresistance. In fact, during the last years, numerous reports indicate that cancer cells resistant to chemotherapy demonstrate significant alterations in mitochondrial metabolism, membrane polarization and mass. Metabolic activity and expression of several mitochondrial proteins are modulated under treatment to cope with stress, making these organelles central players in the development of resistance to therapies. Here, we review the role of mitochondria in chemoresistant cells in terms of metabolic rewiring and function of key mitochondria-related proteins.

## Introduction

Initial studies on cancer metabolism in early 20th century overlooked mitochondria in tumors, as they were deemed dysfunctional^[1]^. Further reports established their critical role in cancer metabolism and, thus, reflected on their key function in disease survival and progression^[2]^. Although traditionally known for their function in cellular bioenergetics coordinating ATP production from cellular respiration, mitochondria play a role in multitude of central cellular activities in physiological and pathophysiological conditions. Indeed, through their multiple regulatory activities, they boost the tumorigenic and metastatic potential in cancer cells^[3-5]^.

On the one hand, they produce precursors for the synthesis of macromolecules such as lipids, proteins, DNA and RNA, which are key to sustain the high proliferative rates of cancer cells. Additionally, mitochondria are central for redox balance: although they are the main producers of reactive oxygen species (ROS) as respiration by-products, mitochondria modulate redox equivalents and gather the majority of enzymatic and non-enzymatic antioxidants^[6]^. The control of redox balance and excessive ROS accumulation is essential for cancer cells, known to present high ROS levels to increase pro-proliferative signaling and activity, and further promoting tumorigenesis through accumulation of new mutations. Apart from ROS, mitochondria also dispose of other cellular waste products, such as ammonia through the urea cycle^[7]^.

Mitochondria provide a common site for multiple metabolic reactions in order to meet energy and biomolecule demands, thus controlling and integrating the diverse metabolic pathways taking place in these organelles. As such, mitochondria enable cancer cells to modulate their metabolism under various stress conditions providing metabolic flexibility^[8-11]^.

Together with cell death regulation, the above-mentioned functions make mitochondria crucial stress sensors, allowing flexibility to adapt to adverse environments such as nutrient deprivation, oxidative stress and DNA damage; all of them present during cancer progression and especially during treatment.

## Drug resistance through rewiring of mitochondrial metabolism

As mentioned above, mitochondria integrate and balance the different metabolic pathways within these organelles, with direct influence on other routes taking place in alternative sites such as the cytoplasm, peroxisomes, *etc*. This metabolic plasticity is a key factor for adaptation to stressful situations, and development of resistance to diverse anticancer drugs.

### Resistance to drugs targeting bioenergetics

Targeting metabolic pathways of cancer has emerged as a promising therapeutic approach. Indeed, myriad metabolic inhibitors have shown encouraging results as anticancer therapeutic agents. However, the emergence of resistant populations, due to metabolic rewiring or intrinsic metabolic heterogeneity found in tumors, highlight some weaknesses in this approach.

Biguanides are a class of well-known metabolic drugs thanks to metformin, which is widely prescribed as anti-diabetic drug. The interest on the antitumoral effects of metformin emerged upon the publication of a retrospective report suggesting that type 2 diabetes patients taking oral metformin showed reduced cancer incidence^[12]^. Metformin has shown promising results against various cancers, such as breast, endometrial and prostate cancer^[13,14]^. Nowadays, it is well accepted that systemic effects on glucose blood levels and direct inhibition of the mitochondrial electron transport chain (ETC) contribute to the antitumoral effects of this drug^[15]^. At the cellular level, metformin treatment induces energetic stress subsequent to reduced ATP production upon ETC blockade, inhibiting proliferation and inducing cell death. Importantly, metformin seems to be especially toxic for cancer stem cells (CSCs), the main tumorigenic and chemoresistant subpopulation found in tumors, due to their dependence on mitochondrial activity^[16,17]^. However, further studies showed that prolonged exposure to metformin gave rise to resistance *in vivo* and *in vitro* in several cancer models, due to the rearrangement of metabolic routes. Although resistance to metformin in pancreatic cancer cells was mainly linked to the ability of cells to increase compensatory glycolysis^[18]^, other studies suggest that mitochondria are able to adapt to the dysfunctional ETC by upregulating alternative mitochondrial pathways. In fact, initial studies on hepatocytes found that metformin treatment induced lipid metabolism^[19]^, guiding subsequent studies in cancer cells. For example, studies conducted on lung cancer cells suggested that metformin treatment altered cellular metabolic phenotype, via AMP-activated protein kinase (AMPK) activation, to reduce dependence on oxidative phosphorylation (OXPHOS) and keep the TCA (tricarboxylic acid) cycle activity for lipid synthesis^[20]^. Under hypoxic conditions, ETC inhibition led to an increased influx of glutamine (Gln) into the TCA cycle^[21]^, highlighting another mechanism used by cancer cells to adapt to suppressed ETC activity. In fact, the susceptibility to metformin of cancer cells, particularly the CSC subset, may be dependent on Gln metabolism: only the combination of metformin with a glutaminase C inhibitor had a substantial CSC suppressing effect^[22]^.

Phenformin, another biguanide targeting complex I of mitochondria, was found to be more potent at inhibiting cell growth in non-small cell lung cancer. However, the emergence of a phenformin-resistant population was observed^[23]^. It has been suggested that the development of resistance is dependent on functional LKB1-AMPK signaling, which promotes a shift in their metabolism to overcome inhibitory effects of phenformin^[23]^. This drug has been withdrawn from the market due to its extreme side effects, although research on phenformin has been reactivated lately due to the mild effects observed upon metformin treatment.

Cancer cells have been found to be dependent on Gln, with a significant upregulation in its uptake^[24,25]^. For that reason, the effects of Gln deprivation or inhibition of the enzymes related to its metabolism has been widely studied *in vitro*. Glutaminase (GA) is a mitochondrial enzyme that converts Gln to glutamate, which is further converted to α-ketoglutarate to fuel the TCA cycle for energy or biomolecule synthesis. GA expression is upregulated in cancers to boost Gln metabolism, and thus has been proposed as an attractive therapeutic target^[26-30]^. GA inhibitors were found to slow tumor growth, increase cell death and sensitize chemotherapy-resistant cells^[31-33]^. Along with drugs, interference RNA is a strategy used to inhibit activity of glutaminase^[34,31]^. However, Gln-dependent cancer cells were able to adapt to GA knockdown by increasing pyruvate carboxylase activity^[35]^, allowing glucose-derived pyruvate to be shunted into the TCA cycle through oxalacetate.

Gln-addicted cells may also be dependent on Gln synthetase (GS) to cover their Gln needs: this enzyme catalyzes the synthesis of Gln from glutamate and ammonia. *De novo* synthesis of Gln plays an important role in cancer survival and adaptation to altered cellular needs and, consequently, many cancer cells upregulate the expression of this enzyme^[36-38]^. Diverse studies underlined the importance of GS in the development of resistance to Gln deprivation. In fact, GS knockdown inhibited the growth of sarcoma cell lines in Gln-deprived media, but had no effect in media supplemented with the aminoacid^[37]^. Importantly, GS overexpression may confer resistance to Gln deprivation in a non-autonomous fashion: luminal breast cancer cells overexpressed GS that, not only allowed them to grow without Gln, but also supported the growth of basal cancer cells in co-culture^[39]^. On the other hand, these studies suggest that cancer cells may develop resistance to anti-GA therapy by increasing *de novo* Gln synthesis via GS to meet their needs, and inhibition of GA and GS together would be necessary to overcome resistance.

Lipid metabolism is frequently altered in cancer cells to meet energy demands, synthesize cellular membranes and mediate cell signaling^[40-42]^. Indeed, cancer cells largely rely on *de novo* lipid synthesis by overexpression of fatty acid synthase (FASN)^[43]^ making FASN inhibition an attractive therapeutic approach with remarkable anticancer activity^[44-46]^. However, studies in colorectal cancer suggest development of resilience to FASN inhibitors^[47]^. Although some evidence points towards altered signaling of metabolism-related kinases (AKT and AMPK) for development of resistance to FASN inhibitors^[47]^, metabolic plasticity conferred by mitochondrial lipid metabolism constitutes the strongest candidate^[40,42]^. Furthermore, cancer cells are also efficient in taking up fatty acids from the microenvironment, another reason FASN inhibition alone may only target a small subset of cells and combination therapies would be necessary^[48]^.

### Resistance to drugs targeting signaling

BRAF is a common oncogene in many cancers, conferring constitutive activation of proliferation pathways. For that reason, BRAF inhibitors have been extensively studied, showing promising results against various cancer types, especially melanoma. However, the emergence of resistance to BRAF inhibitors is a major challenge associated with these drugs. Among the various proposed mechanisms for drug resistance^[49]^, mitochondrial metabolism seems to play a key role. On the one hand, resistant melanoma cells demonstrated an increment of mitochondrial respiration conferred by increased expression of ETC genes^[50]^ and densification of mitochondrial cristae^[51]^. In addition, they showed a high dependency on mitochondrial metabolic activity, maintained on withdrawal of the drug, making them sensitive to mitochondria-targeting compounds in combined therapy^[51]^. On the other hand, BRAF inhibition with vemurafenib led to an increase in Gln uptake consequence of Gln synthesis inhibition^[50]^. For that reason, resistant cells were found to be dependent on mitochondrial Gln, constituting an important vulnerability of these cells^[52]^.

The tyrosine kinase inhibitor imatinib is used mainly to treat chronic myeloid leukemia. This anticancer agent has also been linked to the development of resistant populations. Contrary to BRAF inhibition-resistant cells, the development of resistance to imatinib in various cancers has been linked to increase in glycolysis rather than mitochondrial metabolism^[53]^. Nevertheless, chemoresistance originates in the mitochondria: mitochondrial malfunction in imatinib-resistant cells promote the accumulation of TCA cycle intermediates and NADH, as well as increased ROS generation, as compared to sensitive cells. Interestingly, ROS accumulation was found to be the primary cause of resistance to this drug^[54]^.

### Resistance to other common chemotherapeutic agents

The antimetabolite 5-fluoro-uracil (5FU) has been extensively used as anticancer treatment for a long time. A major issue with this drug, however, is the development of resistance leading to limited clinical use. Among the studies tackling the role of mitochondria on resistance to 5FU, most of them carried out in colon cancer cells, we can identify two opposing mechanisms: resistance through either Warburg-related features or OXPHOS-dependency. On the one hand, increased glucose uptake and metabolism, coupled to mitochondrial dysfunction, has been traditionally considered a hallmark of the Warburg effect. Related to this, it was found that the glucose transporter GLUT1 inhibitor resensitized resistant cancer cells to 5FU^[55]^. Additionally, other studies linked the development of resistance to downregulation of mitochondrial ATP synthase activity^[56]^, which altered cellular bioenergetics and increased glucose dependency. On the other hand, resistance to 5FU has been related to increased mitochondrial mass and activity, amplified expression of ETC enzymes and higher rates of oxygen consumption^[57,58]^. Interestingly, due to their OXPHOS-dependency, resistant cells were found to be sensitive to Complex I inhibition by metformin^[57]^. Strikingly, OXPHOS involvement in resistance to 5FU was linked to the development of stemness-related features, directly connecting CSCs to mitochondrial metabolism as suggested previously^[59,60]^. In line with this, tumor-associated mesenchymal stem cells conferred chemoresistance to 5FU by promoting stemness in gastric cancer cells via mitochondrial fatty acid oxidation (FAO) activation^[61]^. In fact, treatment with the FAO inhibitor etomoxir was able to partially mitigate resistance to 5FU^[61]^.

The development of resistance to the alkylating agent cisplastin has been associated with a metabolic shift from glycolysis to OXPHOS in adenocarcinoma and hepatoma cells^[62]^. The rewiring of cellular metabolism in those tumors was controlled by SIRT1, a master regulator of cellular functions with important implications on metabolism^[62]^. Interestingly, cisplastin resistance in ovarian cancer cells has been also linked to altered metabolism and increased dependency on OXPHOS-generated energy^[63,64]^. In fact, treatment with the Bcl-2 proteins inhibitor ABT-737 was able to reverse cisplastin resistance, possibly by targeting mitochondrial metabolism^[63]^. Additionally, the FAO inhibitor perhexiline also sensitized cisplastin-resistant ovarian cancer cells^[65]^.

Resistance to the antimicrotubule agent paclitaxel has also been associated to mitochondrial metabolism, although in this case independently of glucose. In fact, chemoresistant cells showed a distinct metabolic profile from their sensitive counterparts and their proliferation was inhibited on treatment with FAO inhibitors like mercaptoacetate and etomoxir^[66]^.

The cell cycle inhibitor Cytarabine is a commonly administered chemotherapeutic drug for leukemias and lymphomas, with reduced clinical implications due to development of resistance^[67,68]^. Among the possible mechanisms, Cytarabine treatment favored the expansion of a chemoresistant leukemic stem cell subset with high FAO/OXPHOS activity. Accordingly, the FAO inhibitor etomoxir repressed oxygen consumption in acute myeloid leukemia cells and sensitized cells to cytarabine^[69]^.

## Drug resistance conferred by mitochondrial proteins

Besides metabolism, mitochondria contribute to drug resistance through specific mitochondrial proteins allowing for survival and proliferation under treatment. In fact, the key role of mitochondria in drug resistance has been traditionally attributed to some of the proteins discussed below.

### Uncoupling proteins

Uncoupling proteins (UCPs) have been found to be overexpressed in different cancers and affect mitochondrial potential and ROS levels^[70-72]^. UCPs are inner mitochondrial membrane proteins that provide an alternative path for proton entry into mitochondrial matrix, thereby leading to a reduced electric gradient across the membranes. As the name suggests, they uncouple ATP formation from the ETC and promote the free flow of protons across the membrane, thus leading to a decrease in membrane potential and ROS formation^[73]^.

UCPs have been found to have major implications in cancer and are associated with metabolic reprogramming and therapy resistance^[74,75]^. As mentioned above, these proteins reduce the coupling between two necessary pathways, namely: energy and biomolecule generation pathways. In fact, it has been proposed they modulate cellular metabolism to deal with stress^[76]^. In cancer cells, UCP2 expression was found to promote glycolysis^[77]^ and fatty acid metabolism^[78]^, thus playing a major role in metabolic plasticity^[79]^. On the other hand, UCP2-expressing cells demonstrated better tolerance towards oxidative stress^[80]^, which is key in chemoresistance. For instance, UCP2 expression was found to reduce superoxide production by gemcitabine to produce a protective effect on pancreatic cancer cells, which was reverted by UCP2 inhibition with genipin^[81]^. Similar results were found in human leukemia, where drug-resistant cells overexpressed UCP2 and genipin sensitized these cells to epirubicin and doxorubicin by increasing intracellular ROS^[82]^. Further, due to decrease in ROS formation, UCPs also promote the antiapoptotic activity of cells by inhibition of cytochrome c release and increasing antiapoptotic protein expression^[83-85]^.

### Drug transporters

Several drug transporters are expressed on mitochondrial membrane and their expression is modulated by mitochondrial activity, further contributing to developing resistance by excessive drug efflux^[86,87]^. ATP-binding cassette (ABC) is a super family of transporters known to be responsible for drug efflux and, therefore, drug resistance^[88]^. In fact, drug-resistant cancer cells, particularly CSCs, are found to overexpress ABCs^[89,90]^. Under oxidative stress, cells elevate the expression of various ABC transporters in order to facilitate the entry of glutathione to mitigate it^[91]^. For that reason, it has been suggested that ABC expression is higher in cancer cells which are mitochondria-dependent (including CSCs): they balance their oxidative state controlling ETC-produced ROS, while mitochondrial activity would provide the substrate (ATP) required for ABC activity^[91,92]^.

It is well known that many DNA-targeting chemotherapeutic agents used in cancer induce drug resistance through upregulation of multidrug resistance (MRD) associated genes and multidrug resistance associated proteins (MRP), members of the ABC family of transporters. This was initially described for etoposide-resistance in ovarian cancer cells, in which MRPs allowed efflux of the drug, thereby disallowing its activity^[93]^. A similar mechanism was found in breast cancer cells where MRP expression promoted efflux of the drug and reduced its cellular activity^[94]^. Interestingly, some families of ABCs, including MRPs, can be expressed on mitochondrial membranes, mediating drug resistance by either altering drug transport in order to protect mtDNA (mitochondrial DNA) or inhibiting mitochondrial outer membrane permeability, thus blocking apoptosis^[86]^. In fact, doxorubicin resistance in melanoma cells was associated with expression of ABCB8, a member of ABC transporter family expressed on the inner mitochondrial membrane: ABCB8 overexpression provided doxorubicin resistance protecting mtDNA from damage, while its knockdown resensitized cells to doxorubicin^[87]^. On the other hand, resistance to the topoisomerase inhibitor mitoxantrone could be induced by overexpression of ABCG2 on the mitochondrial membrane, increasing the efflux of the drug^[95]^. The study of mitochondrial drug transporters on therapy resistance is still elusive and needs to be explored further.

### Mitochondrial apoptotic proteins

The role of mitochondria and mitochondrial proteins in apoptosis has been extensively studied and reviewed^[96,97]^. Anti-apoptotic proteins are overexpressed in cancer, making them an important contributor in the development of resistance^[98-101]^.

Cancer cells are enriched in antiapoptotic proteins that inhibit the activity of proapoptotic counterparts^[102]^. In fact, the prosurvival Bcl-2 proteins bind proapoptotic proteins to maintain the mitochondrial membrane potential and inhibit the release of cytochrome c from mitochondria, thus evading apoptosis^[102,103]^. Bcl-2 is upregulated in chemoresistant cells from small cell lung cancer, prostate cancer, ovarian and breast cancer^[63,103-106]^. Similarly, Bcl-xL and Bcl-w, also members of Bcl-2 pro-survival family, are overexpressed in some chemoresistant cancer cells^[63,107]^. Importantly, the upregulation of Bcl-2 expression enabled the development of cross-resistance to chemotherapeutic drugs^[103]^.

Interestingly, alternative mechanisms have been described to favor an antiapoptotic balance in chemoresistant cancer cells. In fact, cells with depleted mtDNA accumulated unprocessed caspases (necessary for apoptosis), which has been suggested as one of the resistance mechanisms^[108]^. Further, loss of signaling metabolites in cancer cells upregulated pro-survival proteins, promoting chemoresistance^[109]^.

Pro-survival Bcl-2 proteins are negatively regulated by BH3-only (BCL-2 homology domain only) proteins. This has led to the development of small inhibitory molecules called “BH3 mimetics”^[110,111]^. These small molecules successfully sensitized chemoresistant cancer cells to treatment^[100]^. For example, the Bcl-2 antagonist ABT-263 induced mitochondria-dependent apoptosis, enhancing activity of chemotherapeutic agents to inhibit small cell lung carcinoma and lymphoid malignancies *in vitro* and *in vivo*^[112]^. Similarly, the Bcl-2 inhibitor ABT-737 antagonized Bcl-2 prosurvival proteins to induce the regression of solid tumors^[113]^. ABT-737 was able to induce significant cytotoxicity in ovarian cancer cells by inhibiting mitochondria respiration and thus sensitized therapy-resistant ovarian cancer cells to chemotherapy. Interestingly, the FAO inhibitors etomoxir and ranolazine activated Bak, a proapoptotic member of the Bcl-2 family, enhancing ABT-737 pro-apoptotic activity in leukemia^[114]^. Obatoclax, another BH3 mimetic, was found to be effective in targeting chemoresistant cells, as well as increasing efficiency of chemotherapeutic drugs^[115]^.

### PGC-1α and mitochondrial biogenesis

Along with altered mitochondrial bioenergetics, cancer cells may increase their mitochondrial mass to rescue them from energy scarcity, and promote survival, growth and propagation^[116-120]^. The main regulators of mitochondrial biogenesis are the PGC-1 [PPAR (peroxisome proliferator-activated receptor)-γ coactivator] family of transcription factors, expressed in energetic cells^[121,122]^. PGC-1 family members, particularly PGC-1α, have been found to be overexpressed in several cancer types, as well as CSC subsets^[18,123-126]^, contributing to tumorigenesis and metastasis.

Importantly, PGC-1α upregulation and enhanced mitochondrial biogenesis are important contributors of chemoresistance development^[127-131]^. In fact, cancer cells alter PGC-1α expression in response to therapy-induced stress, leading to the development of resistance in various ways.

On the one hand, DNA-targeting anticancer agents promote mitochondrial biogenesis^[132,133]^, a process controlled by AMPK-dependent PGC-1α upregulation in HeLa cells^[134]^. Indeed, cells resistant to these agents showed high mitochondrial mass that provides protection against DNA damage^[63,135]^.

PGC-1α induction may also confer chemoresistance by promoting a metabolic shift to overcome ATP requirements, as described for cells treated with BRAF inhibitors (Haq *et al*.^[136]^ 2013). This is also the case for 5FU resistance, which enhanced PGC-1α expression and consequently altered cellular metabolism to mitigate the energetic stress induced by treatment^[58,102,131]^.

Finally, it is important to highlight the regulatory role of PGC-1α and mitochondrial mass for CSCs^[18,135,137]^, the main subpopulation responsible for chemoresistance. For instance, PGC-1α silencing sensitized chemoresistant stem-like cells to chemotherapy^[127]^.

These reports suggest that PGC-1α targeting could help to overcome therapeutic resistance. Although no specific inhibitors of PGC-1α are available to date, indirect inhibition through upstream modulators may be possible. In fact, MAPK inhibitor-resistant melanoma cells were sensitized to therapy when treated with mTORC inhibitors, which decreased PGC-1α expression and reversed metabolic changes^[128]^.

## Conclusion

**Figure 1:**
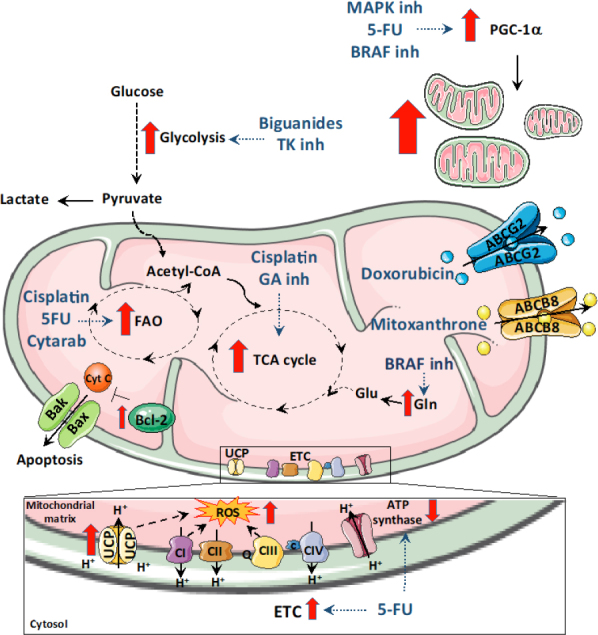
Overview of the mitochondrial determinants of resistance. Some therapeutic drugs promote a shift in metabolism. Drugs like biguanides that target the complex I of mitochondria promote a shift to glycolysis to meet ATP needs. Similarly, glutaminase inhibitors promote cells to switch to oxidative phosphorylation and BRAF protein inhibitors push cells to metabolize glutamine. Mitochondrial FAO contributes to chemoresistance to 5-fluoro-uracil (5FU), Cisplatin and Cytarabine. Increased intracellular ROS induced by enhanced expression of uncoupling proteins (UCPs), electron transport chain (ETC) activity or drug action are also responsible for chemoresistance. In addition to metabolic shifts, therapeutic drugs also elevate the expression of mitochondrial ABC transporters proteins that allow drug efflux and helps to minimize the inhibitory activity of the drug. Bcl-2 family of prosurvival proteins block apoptosis induced by chemotherapeutic drugs. Mitochondrial biogenesis is also induced during therapy-induced stress by elevation of PGC1α

Contrary to the traditional view, mitochondria are now considered the “Achilles heel” of cancer due to the heavy dependency of cancer cells (especially CSCs) on both mitochondrial metabolism and ability to cope with stress. In fact, these mitochondrial properties allow cancer cells to develop resistance to various therapies [Figure 1], suggesting that multimodal therapies targeting mitochondria would constitute promising therapeutic strategies^[33,138,139]^.

However, much research is still needed to unveil the role of mitochondria in cancer. In fact, tumor metabolism may not only change with disease progression and course of treatment: due to intrinsic heterogeneity, mitochondrial function may be variable across tumors and cell subpopulations within a tumor. In fact, it is well known that the activity of mitochondria is largely dependent on various factors, including physical location, nutrient and oxygen availability and the microenvironment. Indeed, cancer-associated cells also exhibit different metabolic phenotypes and directly affect cancer cells metabolism, adding an extra layer of variability. Therefore, carefully designed therapies targeting diverse populations and considering the tumor microenvironment will be crucial in order to develop successful treatment strategies.

## Declarations

### Copyright

© The Author(s) 2019.
